# Vitamin C–squalene bioconjugate promotes epidermal thickening and collagen production in human skin

**DOI:** 10.1038/s41598-020-72704-1

**Published:** 2020-10-09

**Authors:** R. Gref, C. Deloménie, A. Maksimenko, E. Gouadon, G. Percoco, E. Lati, D. Desmaële, F. Zouhiri, P. Couvreur

**Affiliations:** 1 Institut des Sciences Moléculaires d’Orsay, UMR 8214 CNRS, Université Paris-Saclay, 91405 Orsay, France; 2 UMS-IPSIT, UFR de Pharmacie, Université Paris-Saclay, 5 rue Jean-Baptiste Clément, 92296 Châtenay-Malabry, France; 3 UMR/CNRS 8612 Institut Galien Paris-Saclay, UFR de Pharmacie, Université Paris-Saclay, 5 rue Jean-Baptiste Clément, 92296 Châtenay-Malabry, France; 4 UMR-S 999, LabEx LERMIT, Centre Chirurgical Marie Lannelongue, 133 avenue de la Résistance, 92350 Le Plessis-Robinson, France; 5 Laboratoire BIO-EC, 91160 Longjumeau, France; 6 GENEX, 91160 Longjumeau, France

**Keywords:** Chemical modification, Drug delivery

## Abstract

Vitamin C (Vit C) benefits to human skin physiology notably by stimulating the biosynthesis of collagen. The main cutaneous collagens are types I and III, which are less synthesized with aging. Vit C is one of the main promotors of collagen formation but it poorly bypasses the epidermis *stratum corneum* barrier. To address this challenge, we developed a lipophilic version of Vit C for improving skin diffusion and delivery. Vit C was covalently conjugated to squalene (SQ), a natural lipid of the skin, forming a novel Vit C–SQ derivative suitable for cream formulation. Its biological activity was investigated on human whole skin explants in an ex vivo model, through histology and protein and gene expression analyses. Results were compared to Vit C coupled to the reference lipophilic compound palmitic acid, (Vit C–Palmitate). It was observed that Vit C–SQ significantly increased epidermal thickness and preferentially favored collagen III production in human skin after application for 10 days. It also promoted glycosaminoglycans production in a higher extent comparatively to Vit C–Palmitate and free Vit C. Microdissection of the explants to separate dermis and epidermis allowed to measure higher transcriptional effects either in epidermis or in dermis. Among the formulations studied, the strongest effects were observed with Vit C–SQ.

## Introduction

Vitamin C (Vit C) or L-ascorbic acid has important physiologic effects on skin, including promotion of collagen biosynthesis, inhibition of melanogenesis, prevention of radiation-induced damages and acceleration of wound healing^1–3^. Bioavailability of Vit C in skin is inadequate when administered orally^4^. Therefore, the topical route is used to deliver Vit C for local application to promote surgical healing and better tissue reconstruction^4,5^. The use of topical Vit C is preferred in the practice of dermatology^5–8^. Topical administration of Vit C was shown to help with burn wound healing in two ways. It promotes the formation of collagen in skin tissues and removes free radicals as an antioxidant which leads to further improvements at the site of the burn wound^8^.

Incubation of cultured skin substitutes in media containing Vit C results in several benefits such as greater viability and more complete basement membrane development. It was suggested that Vit C improves anatomy and physiology of cultured skin substitutes and promotes cellular viability and formation of epidermal barrier in vitro^9^.

Collagen is the most abundant protein produced by mammals and it is fundamental in the constitution of a contiguous interstitium throughout the epidermis. Type I and III collagens are formed in human skin in a higher proportion relative to other types and are maintained in a fixed proportion relative to one another in normal skin tissue^10^. They are the main constituents of the dermal extracellular matrix and play a major role in skin elasticity and aspect. However, type I and III collagen respective contents and distribution in skin vary as a function of age. In studies involving skin from donors with ages < 18 up to > 50, it was shown that the mean content of type I and III and type I/III collagen ratio in skin differed significantly among age groups (*p* < 0.05), with the lowest levels of type I, III, and the highest ratio of type I/III observed in the elderly age group^11,12^. It was concluded that the amount of collagen III significantly diminishes with age^13^.

To exert its beneficial action on collagen biosynthesis, Vit C needs to bypass the skin natural barrier. The *stratum corneum* (SC) provides the principal obstacle that limits the percutaneous penetration of topically applied Vit C^14,15^. Controlled laser ablation of SC was found to improve Vit C penetration into the skin^16^, but this treatment needs to be performed under strictly controlled conditions. Thus, there is an evident interest to develop an efficient Vit C formulation able to penetrate through skin without the need of lasers or other physical treatments, allowing Vit C to promote epidermal thickness and collagen production^17^. Different approaches were carried on to modify Vit C with lipid moieties in an attempt to improve its skin penetration. Common topical formulations of lipid conjugates of Vit C include ascorbyl-6-palmitate, disodium isostearyl 2-*O*-ascorbyl phosphate and tetraisopalmitoyl ascorbic acid^18,19^. However, in some cases, a daily application of ascorbyl-6-palmitate, and other ascorbic acid derivatives did not increase the levels of L-ascorbic acid in the skin^20^. On the other hand, clinical studies on the efficacy of topical formulations of Vit C remain limited, and the challenge lies in finding the most stable and permeable formulation in achieving the optimal results^21^. The squalenic acid chain with its 27 carbon atoms gives an ascorbyl conjugate endowing high hydrophobicity (LogP = 7.08, MarvinSketch) that appeared much more suitable for skin penetration than the corresponding palmitic or disodium isostearyl 2-*O*-ascorbyl phosphate (LogP = 5.01 and 5.30 respectively). Furthermore, ascorbyl conjugates of (*Z,Z*)-1,4-polyunsaturated lipid acids such as linoleic acid or DHA suffer from a poor chemical stability.

In this sense, squalene (SQ) is a highly stable polyisoprenyl compound with robust (*E,E*)-1,5-trisubstituted double bond systems and appears as a good candidate for Vit C modification. It was our idea that SQ, a main component of sebum^22^ and naturally present on the skin, could confer a lipophilic character to Vit C by promoting a better diffusion and interaction with the skin. To achieve this challenging goal, Vit C was covalently conjugated to squalene (SQ), forming a novel Vit C–SQ bioconjugate. Vit C coupled to another lipophilic compound, palmitic acid, (Vit C–Palmitate) was used as a reference. These various Vit C formulations were compared for their biological activities upon human whole skin explants in an ex vivo model. Histological, as well as, biomolecular approaches at protein and gene expression levels were used to compare the biological effects of these Vit C formulations.

Here we show that Vit C–SQ significantly increased epidermal thickness and preferentially favored collagen III production in skin human explants after application for 10 days. The novel bioconjugate also promoted glycosaminoglycans (GAGs) production to a higher extent than Vit C–Palmitate. The transcriptional effects were found to be higher either in epidermis or in dermis, according to the considered genes. Among the formulations studied, the strongest effects were observed with Vit C–SQ complex, confirming the improvement of the physiological functions of the skin observed with this bioconjugate.

## Results and discussion

### Synthesis and chemical characterization of Vit C–SQ

**Figure 1:**
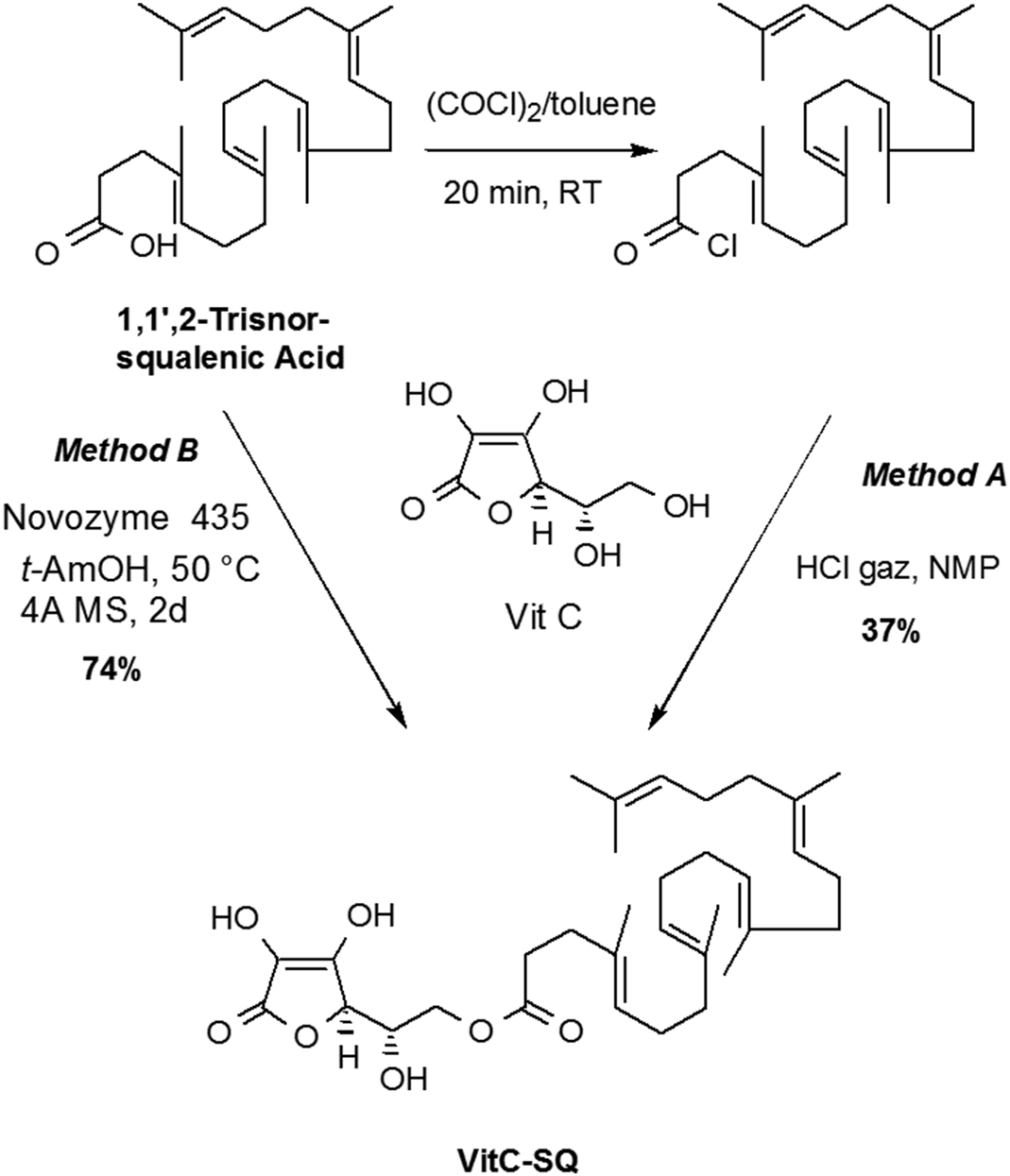
Synthesis of Vit C–SQ through chemical esterification (Method A) or enzymatic acylation (Method B).

Vitamin C (ascorbic acid) is a charged and hydrophilic natural antioxidant with skin antiaging and photoprotective effects. Because it penetrates poorly into the skin, lipidic derivatives more able to enter *stratum corneum* have been designed as antioxidant excipient for topical formulations. Many fatty acids esterified forms of the C-6 primary hydroxyl group of vitamin C were thus introduced in pharmaceutical or cosmetic compositions. For example, 6-O-palmitoyl-L-ascorbic acid was shown to reduce the appearance of wrinkles, cracks, crevices and puffiness around the eyes^23^ and 6-*O*-stearoyl-L-ascorbic acid was found to retard skin aging^24^ and to be a potent inhibitor of hyaluronidase enzymes^25^. Many other derivatives including esters of oleic acid^26^, linoleic^27^, myristic acid^28^, retinoic acid^29^, docosahexaenoic acid^30^, etc. have been evaluated for potential application in cosmetic and as antioxidant in food industry. The anchoring of the lipidic moiety on the lateral chain of ascorbic acid preserves the redox properties of the tetronic core. Furthermore, this design considerably simplifies the synthesis since the primary hydroxyl group is the most nucleophilic function. We thus envisaged to access 6-squalenoyl ascorbic acid (Vit C–SQ) by direct condensation of 1,1′,2-trisnor-squalenic acid, easily available from squalene^31,32^, without protection of the tetronic ring. Several synthesis strategies were described to obtained SQ derivatives^33–35^. However, in the case studied here, most conventional methods failed to give the expected material. We finally found that when ascorbic acid was treated with squalenyl chloride in HCl saturated *N*-methyl-pyrrolidinone (NMP) according to the method of Yazawa et al.^36^, the desired 6-*O*-squalenoyl-L-ascorbic acid could be obtained in 30–35% yield after chromatographic purification. Although this method had the merit of giving the desired product, it suffered from extensive degradation during the removal of the high boiling solvent NMP. To bypass this hurdle, we examined the enzymatic acylation of ascorbic acid with stabilized lipase. Biocatalysts offered mild conditions and improved regioselectivity for the acylation of ascorbic acid, particularly when sensitive unsaturated fatty acids were involved^37–39^. The 6-*O*-squalenoyl-L-ascorbic acid was thus synthetized by treatment of a 2:1 mixture of ascorbic acid and squalenic acid with Novozyme 435, a lipase acrylic resin from *Candida antarctica* in *t-*AmOH at 50 °C for 2 days, using molecular sieves as drying agent. In these conditions the Vit C–SQ was obtained in 74% yield as a thick oil after chromatographic purification (Fig. 1). The Vit C–SQ derivative was stable upon 2 years storage in a freezer at − 20 °C.

### Formulation of Vit C–SQ

Vit C–SQ and Vit C–Palmitate (used as a control) were formulated as creams to be directly applied on human explants. These oily compounds were dispersed in SQ at equivalent concentrations of Vit C (3 or 5 wt%) and homogenized by magnetic stirring. The Vit C–SQ and Vit C–Palmitate formulations were viscous, with an oily texture and slightly yellow color. Free Vit C could not be dispersed in SQ, so it was incorporated in carboxymethyl cellulose at equivalent contents.

### Skin morphology was improved by Vit C–SQ bioconjugate

Formulations containing free or conjugated Vit C or SQ alone, were applied repeatedly for 10 days upon *stratum corneum* of human skin explants collected from two 30-year females and maintained in a proprietary survival medium. Untreated control explants from each donor were kept in the same conditions for the same time. The explants from both donors received distinct treatment panels, i.e. donor 1 received for 10 days Vit C either free or coupled to SQ or to Palmitate, at 1 or 3  wt% final Vit C concentration, while donor 2 received for 10 days free Vit C 5 wt% or Vit C–SQ at 3 or 5 wt%, or SQ alone. Thus, the experiment with Vit C–SQ 3 wt% was repeated in both donors, allowing comparison.

**Figure 2:**
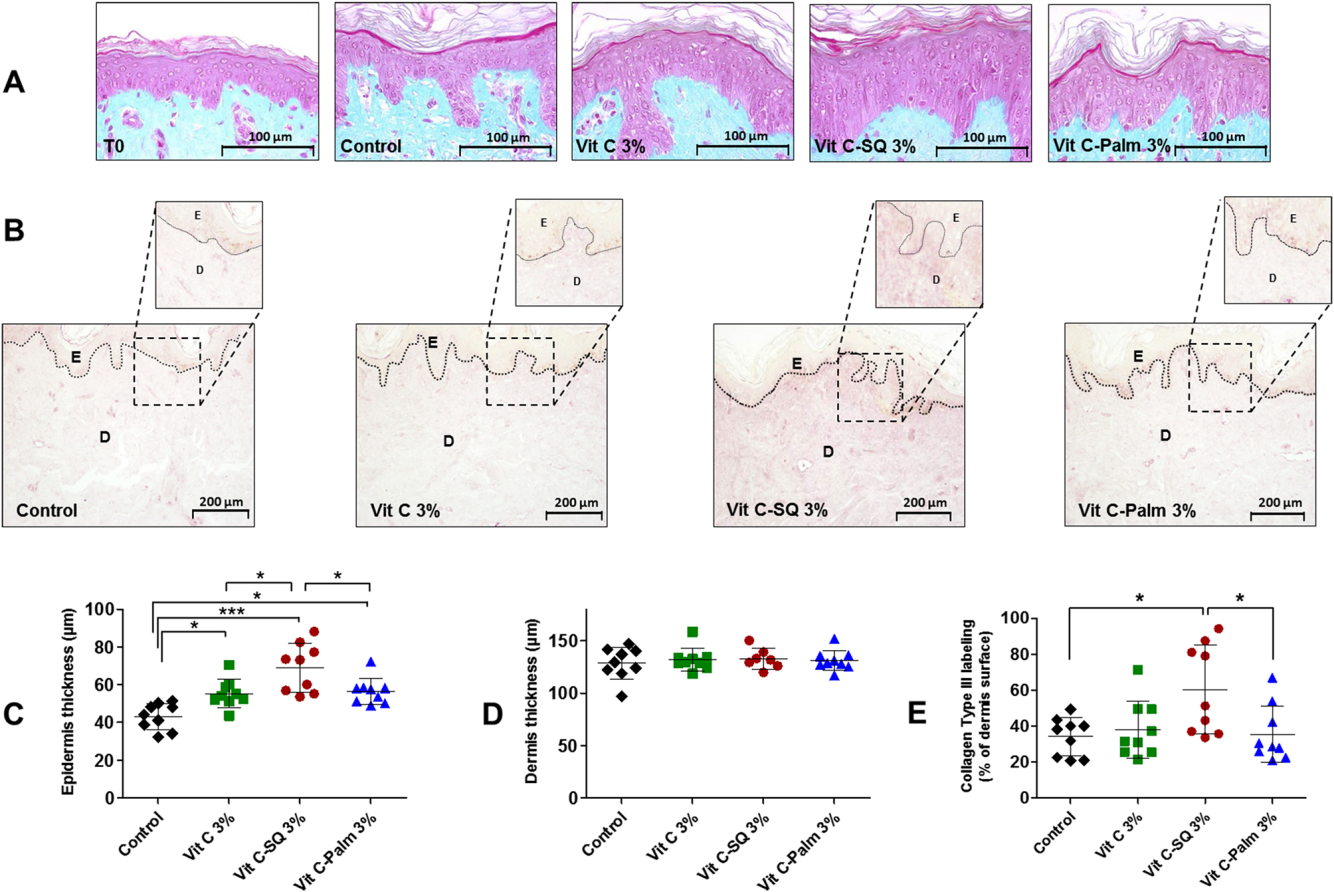
Topical treatment of human skin explants (donor 1) for 10 days with three formulations containing 3 wt% Vit C, increased epidermis thickness and collagen type III labeling in dermis. (**A**) In fixed and stained (Masson trichrome) whole skin sections, epidermis (pink) and dermis (blue) layers were observed before (T0) and after the treatment period. The control explant was kept untreated for 10 days. (**B**) Fixed skin sections labeled with goat anti-human collagen III antibody and revealed by DAB are shown. Pictures were realized with a tri CCD DXC 390P camera (Sony) and stored with Leica IM1000 software, version 1.10 (www.leica-microsystems.com). (**C**) The epidermis thickness and (**D**) dermis thickness were measured in each explant (n = 7 to 9 images; mean of 3 measures per image). (**E**) The intensity of collagen III immuno-staining signal in dermis was measured as the percentage of analyzed surface (n = 9 images; mean of 3 measures per image). One-way ANOVA with Tukey’s post-test (***p < 0.001, *p < 0.05) was used to compare treatments. Graphs were done with GraphPad Prism version 5.0.0 for Windows (GraphPad Software, San Diego, California USA, www.graphpad.com). Scale bar = 100 µm (panel A), and 200 μm (panel B).

In fixed and colored tissue sections from each explant, the skin morphology as well as the epidermis and dermis thickness were investigated. A significant increase of epidermis thickness was observed with Vit C–SQ 3 wt% in donor 1 (Fig. 2A, C), i.e. + 60% compared to the untreated control (69 ± 12 µm versus 43 ± 7 µm, respectively; *p* < 0.001). The thickening effect was more limited with the same concentration of free Vit C (+ 28%; *p* < 0.05) or of Vit C–Palmitate (+ 31%; *p* < 0.05). Consistently, in donor 2 a 22% increase in epidermis thickness was observed with Vit C–SQ 3 wt% (*p* < 0.05), as well as, with Vit C–SQ 5 wt% (*p* < 0.05), while only a non significant 14% increase was observed with free Vit C 5 wt% and no variation with SQ alone (Supplementary Fig. S1A, C), thus confirming the beneficial effect of Vit C–SQ on skin thickness. Epidermal thickness before treatment (40 ± 4 µm) was not significantly different from the value after 10 days without treatment (43 ± 7 µm; T0) in donor 1 (Fig. 2A). On the other hand, dermis thickness (128 ± 14 µm in control from donor 1; 125 ± 4 µm in control from donor 2) was not modified whatever the Vit C formulation used in both donors nor with SQ in donor 2 (Fig. 2D, Supplementary Fig. S1D). Finally, of all Vit C formulations studied, Vit C–SQ resulted in the highest gain of epidermis thickness.

### The skin biological pathways were studied at molecular level

**Figure 3:**
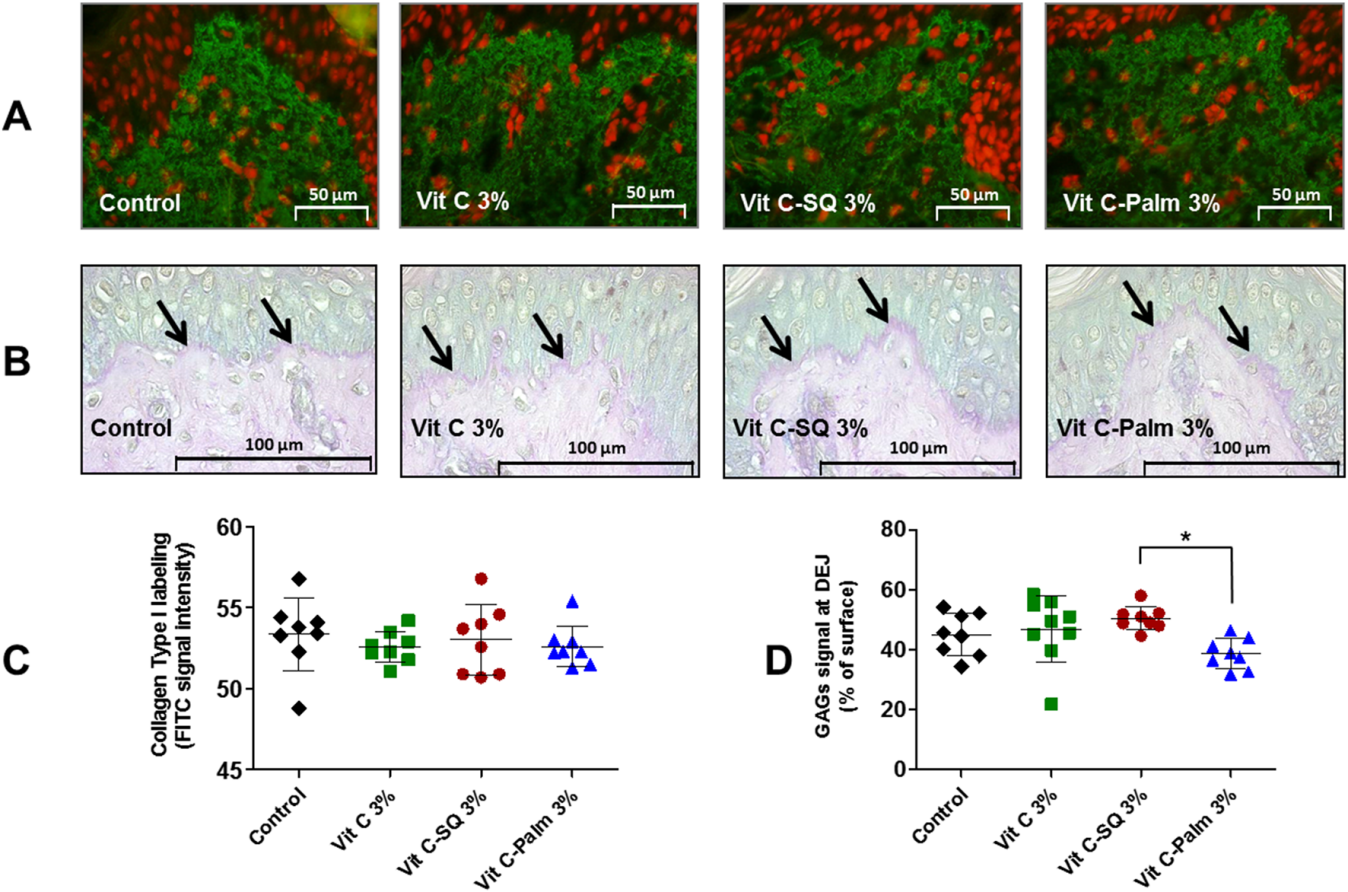
Effects of ex-vivo topical treatment of human skin explants (donor 1) for 10 days with three formulations containing 3 wt% Vit C, upon collagen type I expression in whole skin and GAGs staining at dermal–epidermal junction (DEJ). (**A**) Collagen type I was labeled by a rabbit anti-human collagen I antibody with biotin/streptavidin amplification and FITC fluorescence revelation (green) and cell nuclei stained with propidium iodide (red). Pictures were realized with a tri CCD DXC 390P camera (Sony) and stored with Leica IM1000 software, version 1.10 (www.leica-microsystems.com). (**B**) Staining of fixed skin sections with Schiff reagent revealed the neutral GAGs as purple pink near DEJ (arrows), at the bottom of upper epidermis layer. (**C**) One-way ANOVA with Tukey’s post-test was used to compare collagen I signal (n = 8 images; mean of 3 measures per image), relative to untreated control (not significant). (**D**) GAGs coloration measured as percent of DEJ surface (n = 8 to 9 images; mean of 3 measures per image) from each explant was compared in formulations and untreated control by one-way ANOVA with Tukey’s post-test (*p < 0.05). Graphs were done with GraphPad Prism version 5.0.0 for Windows (GraphPad Software, San Diego, California USA, www.graphpad.com). Scale bar = 50 µm panel A, and 100 μm panel C.

The expression of proteic (collagens; Figs. 2B, 3A) as well as glucidic skin markers (GAGs; Fig. 3B, Supplementary Fig. S1B, E) has been measured by immuno-histochemistry in human tissue explants after the different topical treatments.

To gain insights on the molecular pathways behind the observed effects of Vit C–SQ compared to Vit C–Palmitate, to free Vit C or to SQ, a set of 15 genes of interest were selected according to their known relationships with skin physiopathology, which have been documented using the Ingenuity Pathway Analysis (IPA) software^40^ (Supplementary Fig. S2). The expression of these genes was measured by RT-qPCR in the same skin explants previously treated with the various formulations of Vit C or SQ alone (Supplementary Fig. S3). The expression of some of these genes was also evaluated in epidermal and dermal compartments previously separated by laser capture microdissection. For some genes, the transcriptional effects were higher upon epidermis compared to dermis, which might be related to the epidermis thickening described before (Supplementary Fig. S4).

It can be concluded from this study that the Vit C–SQ complex has modified in the strongest extent the transcriptional expression of most of the target genes studied, in a globally reproducible manner for the Vit C–SQ 3 wt% tested in two independent donors. The effects observed in whole skin were also consistent with the sum of the effects measured in separately microdissected epidermis and dermis in the two donors. Moreover, the transcriptional expression data showed consistency with histological data. Hereafter the effects of this bioconjugate upon the various biological functions explored will be reviewed.

### Vit C–SQ favors biosynthesis and maintenance of structural fibers of the skin

**Figure 4:**
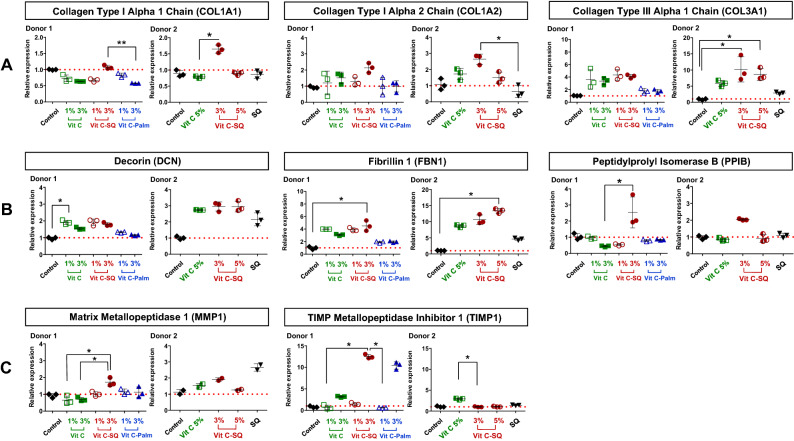
Vitamin C formulations influenced the expression in human skin explants, of the mRNA transcripts of (**A**) genes encoding collagens type I and type III, (**B**) genes controlling skin fibrils assembly and (**C**) genes regulating the extracellular matrix (ECM). Fold changes of each transcript were calculated as 2^−ΔCq^ from Cq normalized by NORMA-gene algorithm^56^, in whole skin explants treated with 1–3 wt% free or formulated Vit C (in donor 1) or with 3–5 wt% free or squalenized Vit C or with squalene (SQ) alone (in donor 2), relative to the untreated control (set to 1, red dotted line). The data from one of two or three analyzed explants are presented (n = 3 per group). Kruskal–Wallis non-parametric test followed by Dunn’s post-test was used to determine significance between the groups (**p < 0.01, *p < 0.05). Graphs were done with GraphPad Prism version 5.0.0 for Windows (GraphPad Software, San Diego, California USA, www.graphpad.com).

Collagen is a major constituent of skin where it is structured into fibers representing the major part of dermis mass^17^. Type III collagen is a homotrimer forming a right-handed triple helix^4^ which acts as a major structural constituent of the extracellular matrix. The immuno-histochemistry studies performed in donor 1 showed that the collagen III protein content was twice higher in papillary dermis after 10-days treatment with 3 wt% Vit C–SQ formulation comparatively to untreated control, i.e. collagen III signal represented 60 ± 23% versus 34 ± 10% of dermis surface, respectively (*p* < 0.05). The more limited effects of Vit C–Palmitate (+ 4%) and of free Vit C (+11%) were not significant (Fig. 2B, E). Consistently, this stimulating effect of collagen III biosynthesis by Vit C–SQ upon was also observed in both donors at gene expression level. The *COL3A1* mRNA transcript was increased in donor 1 (fourfold) and in donor 2 (11-fold; *p* < 0.05) after Vit C–SQ 3 wt% treatment versus control, and similarly with Vit C–SQ 5 wt% in donor 2 (tenfold; *p* < 0.05) (Fig. 4A). Comparatively, the free Vit C produced a smaller and not significant increase in *COL3A1* expression in both donors, while Vit C–Palmitate was almost devoid of any effect in donor 1, as well as SQ alone in donor 2.

The collagen I protein content was not modified in dermis from skin explants treated for 10 days with any of the 3 wt% Vit C formulations tested, according to immuno-histochemistry results in donor 1 (Fig. 3A, C). Among the genes encoding type I procollagen^41^, the expression of *COL1A1* gene, encoding collagen type I alpha 1 chain, was not modified by Vit C–SQ in donor 1 and was poorly raised in donor 2 (1.6-fold, *p* < 0.05). The *COL1A2* gene encoding collagen type I alpha 2 chain was slightly overexpressed after Vit C–SQ 3 wt% treatment in both subjects studied, i.e. 2.1 to 2.6-fold (*p* < 0.05). In contrast, free Vit C, Vit C–Palmitate or SQ alone had no significant effect in collagens I gene expression (Fig. 4A).

Our transcriptional data obtained from skin layers after microdissection suggest that the overexpression of collagens I and III induced by Vit C–SQ could take place in dermis (Supplementary Fig. S4), consistently with described stimulation of collagen production by Vit C in dermis fibroblasts^17^. Overall, among the collagen-encoding genes studied here, *COL3A1* was the most strongly modified and Vit C–SQ was the most active formulation since it stimulated 2–4 times more its expression than that of *COL1A2*. These results are in line with studies on liposomal Vit C showing increased production of collagen in pig ear skin^35^. Our study demonstrates that collagen production is stimulated by Vit C derivatives also in human skin. Finally, the observed effects of Vit C–SQ upon collagens I and III contribute to a limited I / III ratio, which is typical of young skins^11,12^. This could characterize an anti-aging action for this bioconjugate.

Decorin (DCN)^42,43^ and fibrillin 1 (FBN1)^44^ are involved in the assembly and maintenance of collagen fibrils and elastic microfibrils, thus contributing to skin strength and suppleness. The corresponding genes were significantly overexpressed in whole skin explants from both subjects, treated with Vit C formulations, with a higher effect of the Vit C–SQ conjugate, i.e. *DCN* twofold to threefold and *FBN1* fourfold to tenfold (*p* < 0.05), while other formulations had lower or no effect (Fig. 4B). In microdissected skin layers, the transcriptional effect of Vit C–SQ was observed in epidermis for *FBN1*, thus this might be related to the epidermis thickening described above; however, it occurred at a similar extent in dermis and epidermis for *DCN* (Supplementary Fig. S4). Moreover, the gene encoding peptidylprolyl isomerase B (PPIB) which contributes to collagen binding, accelerates proteic folding and favors tensile strength of skin^45^, was twice overexpressed with Vit C–SQ 3 wt% (*p* < 0.05), while it was not affected by free Vit C or Vit C–Palmitate 3 wt% or by SQ (Fig. 4B).

### Vit C–SQ positively regulates the components of ECM

**Figure 5:**
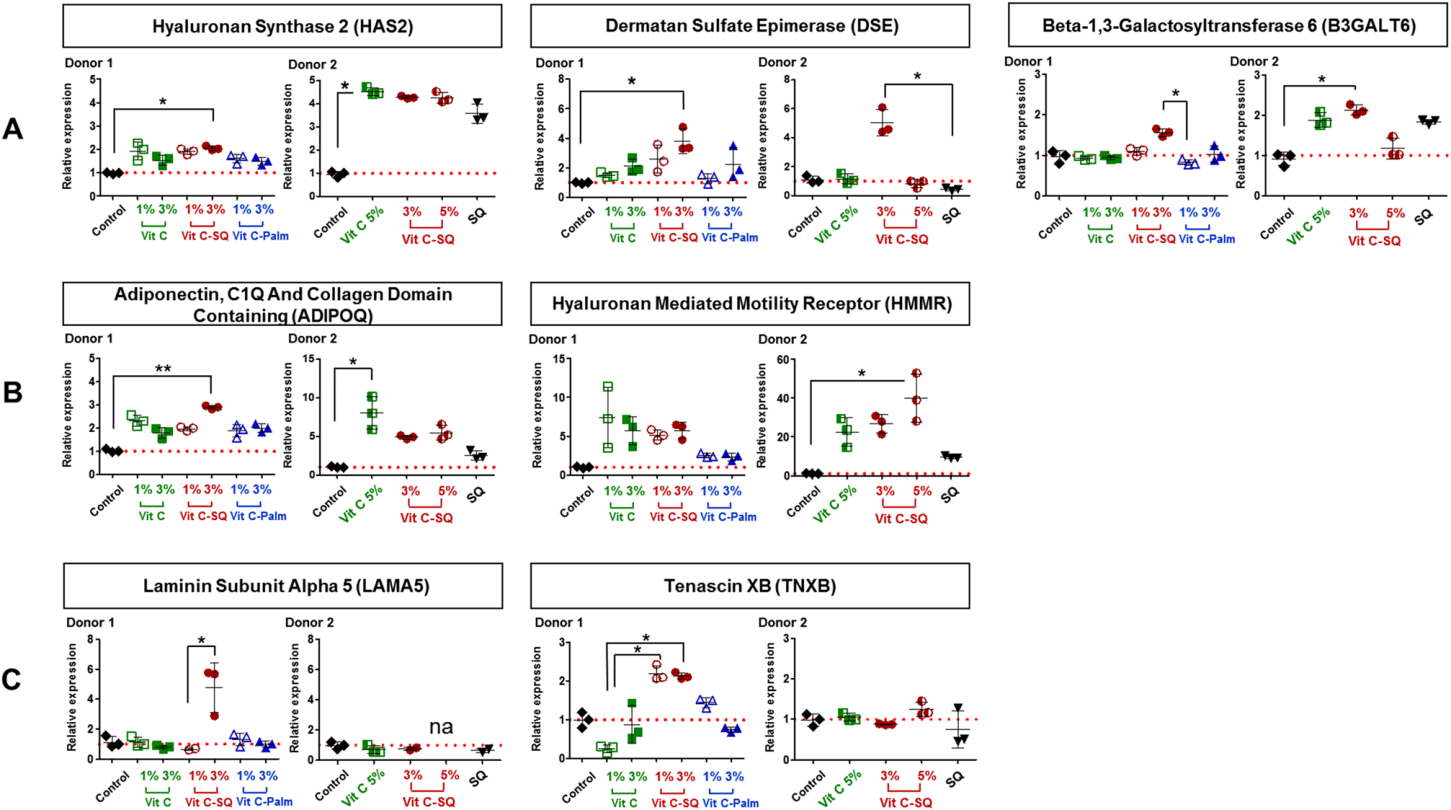
Vitamin C formulations influenced the expression in human skin explants, of the mRNA transcripts of (**A**) genes encoding enzymes of GAGs biosynthesis, (**B**) genes controlling cell growth and motility, and (**C**) genes regulating ECM. Fold changes of each transcript were calculated as 2^−ΔCq^ from Cq normalized by NORMA-Gene algorithm^56^, in whole skin explants treated with 1–3 wt% free or formulated Vit C (in donor 1) or with 3–5 wt% free or squalenized Vit C or with squalene (SQ) alone (in donor 2), relative to the untreated control (set to 1, red dotted line). The data from one of two or three analyzed explants are presented (n = 3 per group). Kruskal–Wallis non-parametric test followed by Dunn’s post-test was used to determine significance between the groups (**p < 0.01, *p < 0.05). Graphs were done with GraphPad Prism version 5.0.0 for Windows (GraphPad Software, San Diego, California USA, www.graphpad.com).

The matrix metalloproteinases (MMPs) are known to contribute to basement membrane and ECM degradation through the fragmentation of collagens, during skin aging and ultraviolet irradiation^46^. In our study, the Vit C–SQ complex stimulated 6-times more in donor 1 the transcriptional expression of TIMP metallopeptidase inhibitor of MMPs (*TIMP1*; 12-fold, *p* < 0.05; Fig. 4C) and twice more the expression of *COL3A1* transcript (fourfold; Fig. 4A), than the expression of *MMP1* transcript (1.7-fold, *p* < 0.05; Fig. 4C) which encodes the collagenase involved in the degradation of type I and III collagens^41^. Such effects can contribute to preserve the existing collagen. This is consistent with the observed favorable action of Vit C–SQ upon the production of collagens I (Fig. 4A) and III (Figs. 2B, E, 4A) and on the expression of decorin (Fig. 4B), which locates at the surface of collagen fibers thus protecting them against cleavage by MMPs^43^. Moreover, members of two families of ECM glycoproteins, i.e. laminins, which are the major non collagenous constituent of basement membranes, and tenascins, involved in wound healing^47^, were also positively modulated at gene level in donor 1 by Vit C–SQ 3 wt% only: laminin subunit alpha 5 (*LAMA5*; fourfold, *p* < 0.05) and tenascin XB (*TNXB*; twofold, *p* < 0.05) (Fig. 5C).

### Vit C–SQ is favorable to GAGs content in skin

Hyaluronic acid (hyaluronan, HA) is an acidic GAG predominantly present in skin where it retains water and promotes cell motility, adhesion, proliferation and tissue organization. Moreover, HA accumulates in ECM in early stages of wound healing^48^. Hyaluronan synthase 2 (HAS2) mediates the biosynthesis of HA^49^ and is described as its main synthase in skin^50^. The biosynthesis of HA could be stimulated by Vit C–SQ 3 wt% in skin, as suggested by the overexpression in whole skin explants, of the *HAS2* gene, i.e. twofold (*p* < 0.05) in donor 1 and fourfold in donor 2 (Fig. 5A).

This is also consistent with the observed overexpression to a similar extent after treatment with Vit C–SQ 3 wt% (threefold, *p* < 0.01 in donor 1), of the gene encoding adiponectin (*ADIPOQ*), which promotes biosynthesis of HA along with *HAS2* transcript^51^ (Fig. 5B). The effect of Vit C–SQ upon this gene supports the observed thickening of the epidermis (Fig. 2A, C), since adiponectin favors cell growth and tissue remodeling through the binding and sequestration of growth factors. The hyaluronan mediated motility receptor (HMMR), which is a major HA receptor and regulates cell growth, motility and contact inhibition^48^ was also strongly overexpressed (sixfold in donor 1; 40-fold, *p* < 0.05 in donor 2) by Vit C–SQ 3 or 5 wt% in whole skin (Fig. 5B).

Moreover, histochemistry staining of neutral GAGs, which act as growth factors reservoirs at the dermal–epidermal junction (DEJ)^48^, showed a stronger signal at DEJ in skin explants treated with Vit C–SQ 3 wt% compared to Vit C–Palmitate 3 wt% in donor 1 (+ 30%, *p* < 0.05; Fig. 3B, D). Consistently, skin explants treated with Vit C–SQ 3 wt% exhibited a 50%-higher GAGs signal at DEJ, compared to free Vit C 5 wt% in donor 2 (*p* < 0.05; Supplementary Fig. S1B, E). The genes encoding two enzymes involved in GAGs biosynthesis were consistently overexpressed in both donors after Vit C–SQ 3 wt% treatment, i.e. beta-1,3-galactosyltransferase 6^52^ (*B3GALT6*; twofold, *p* < 0.05) and dermatan sulfate epimerase^53^ (*DS*E; fourfold, *p* < 0.05) (Fig. 5A).

## Conclusion

Topical application of Vit C on the skin with a strategy for the delivery through the SC has proved its usefulness as a complement to Vit C nutritional intake, as well as, in various cases of skin damage situations, such as UV irradiation, inflammation, redox unbalance, aging and wound healing^4,19^. At equivalent concentrations in Vit C, the Vit C–SQ bioconjugate exerted higher beneficial actions on human skin comparatively to free Vit C or Vit C linked to palmitic acid. Not only Vit C–SQ triggered a marked epidermis thickening, but it also induced the production of collagen III typical of young skins, as well as, the assembly of collagen fibers and elastic microfibrils and favored cutaneous hydration by stimulating production of mucopolysaccharides which have high water retention ability. With further studies, Vit C–SQ based formulations could find potential applications in cosmetics and dermatology care. In a nutshell, the VitC-SQ bioconjugate demonstrated a beneficial action on skin, although its mechanism of action still remains uncovered. It is, however, hypothesized that, apart the preservation of the Vit C anti-oxidant activity through the conjugation to SQ, this lipid may also favor skin penetration and possible interaction with cell membranes.

## Experimental section

### Synthesis of Vit C–SQ

IR spectra were obtained as solid or neat liquid on a Fourier Transform Bruker Vector 22 spectrometer. Only significant absorptions are listed. The ^1^H and ^13^C NMR spectra were recorded on Bruker Avance 300 (300 MHz and 75 MHz, for 1H and 13C, respectively) or Bruker Avance 400 (400 MHz and 100 MHz, for ^1^H and ^13^C, respectively) spectrometers. Recognition of methyl, methylene, methine, and quaternary carbon nuclei in ^13^C NMR spectra rests on the J-modulated spin-echo sequence. Mass spectra were recorded on a Bruker Esquire-LC. Analytical thin-layer chromatography was performed on Merck silica gel 60F254 glass precoated plates (0.25 mm layer). Column chromatography was performed on Merck silica gel 60 (230–400 mesh ASTM). Toluene and N-methyl pyrrolidone (NMP) were distilled from calcium hydride, under a nitrogen atmosphere. *t*-AmOH, was dried of sodium and distilled. All reactions involving air- or water-sensitive compounds were routinely conducted in glassware which was flame-dried under a positive pressure of nitrogen or argon. Ascorbic acid, oxalyl chloride, squalene, NMP and Novozyme 435 (L4777) were purchased from Sigma-Aldrich Chemical Co., France. Chemicals obtained from commercial suppliers were used without further purification. 1,1′,2-tris-norsqualenic acid was prepare according to Ceruti et al.^32^. For comparison purposes, palmitoyl ascorbate was prepared from palmitic acid and ascorbic acid according to litterature^54^.

#### 4,8,13,17,21-Pentamethyl-docosa-4,8,12,16,20-pentaenoyl chloride (2)

A solution of trisnorsqualenic acid (1.80, 4.5 mmol) in anhydrous toluene (10 mL) was degassed by bubbling a stream of nitrogen through the solution for 5 min. Oxalyl chloride (1.74 g, 13.8 mmol) was added dropwise at 20 °C. The reaction mixture was stirred for 3 h at the same temperature and concentrated under reduced pressure to give the title compound as a yellow oil which is used directly in the next step. IR (film) ν: 2920, 1799, 1443, 1382, 955, 893 cm^−1^; RMN ^1^H (300 MHz, CDCl_3_) *δ* : 5.23–5.10 (m, 5 H), 2.45 (t, *J* = 7.3 Hz, 2 H), 2.30 (t, *J* = 7.5 Hz, 2H), 2.15–1.95 (m, 16 H), 1.70 (s, 3H), 1.62 (s, 15H) ppm; RMN ^13^C (75 MHz, CDCl_3_), *δ* :173.2 (C, *C*OCl), 135.1 (C, *C*=CH), 134.8 (C, *C*=CH), 134.6 (C, *C*=CH), 131.4 (C, *C*=CH), 131.1 (C, *C*=CH), 126.6 (CH, C=*C*H), 124.7 (CH, C=*C*H), 124.4 (CH, C=*C*H), 124.3 (2CH, C=*C*H), 45.8 (CH_2_), 39.7 (2CH_2_), 34.4 (CH_2_), 34.6 (CH_2_), 28.2 (2CH_2_), 26.8 (CH_2_), 26.7 (CH_2_), 26.5 (CH_2_), 25.7 (CH_3_, C=C(*C*H_3_)_2_), 17.6 (CH_3_), 16.0 (CH_3_), 15.9 (2 CH_3_), 15.8 (CH_3_) ppm.

#### 6-*O*-[4,8,13,17,21-Pentamethyl-docosa-4,8,12,16,20-pentaenoyl] ascorbic acid, method A

A stream of dry HCl was passed through 5 mL of *N*-methylpiperidone in a washing bottle until the *entire mass solidified. N*-methylpyrrolidinone was added to get a clear solution (~ 5 mL) which was transferred into a 50 mL round bottom flask. Ascorbic acid (0.7 g, 4.0 mmol) was added and the mixture was stirred until complete dissolution. The reaction mixture was cooled to 0 °C and squalenoyl chloride (418 mg, 1.0 mmol) was added. After being stirred for 20 h at room temperature, water was added (20 mL) and the mixture was extracted with ethyl acetate (4 × 20 mL). The combined organic layers were washed with water (2 × 5 mL) dried over MgSO_4_ and concentrated under reduced pressure. The residual NMP was removed under reduced pressure using dry ice rotavapor (60 °C, 0.05 mm Hg,) to leave an oil which was purified by chromatography over silica gel eluting with ethyl acetate and then AcOEt/MeOH (98:2) to give the title compound as a pale yellow thick oil (190 mg, 34%).

#### 6-*O*-[4,8,13,17,21-Pentamethyl-docosa-4,8,12,16,20-pentaenoyl] ascorbic acid, method B

To a suspension of ascorbic acid (200 mg, 2 mmol) in anhydrous *tert*-amyl alcohol (5 mL) was successively added Novozyme 435 (150 mg), tris-norsqualenic acid (200 mg, 1.0 mmol) and 1 g of 4 Å molecular sieves. The flask was fixed to a rotavapor and slowly stirred at 50 °C (water bath). After 48 h, the reaction mixture was cooled and filtered through a plug of Celite. The solid was thoroughly washed with AcOEt and the filtrate was concentrated under reduced pressure. The crude product was purified by chromatography over silica gel eluting with ethyl acetate and then AcOEt/MeOH (98:2) to give the title compound as a pale yellow thick oil (209 mg, 74%); [α]_D_ =  + 10 (c = 0.5, EtOH); IR (film) ν: 3600–2800, 2976, 2857, 1744, 1696, 1443, 1382, 1350, 1296, 1151, 1119, 1043, 983, 844 cm^−1^; NMR ^1^H (360 MHz, MeOH-d_4_) *δ*: 5.20–5.05 (m, 5H), 4.72 (d, *J* = 2.0 Hz, 1H, H-4), 4.24 (dd, *J* = 11.16 Hz , *J* = 6.84 Hz, 1H, H-6), 4.18 (dd, *J* = 11.16 Hz , *J* = 6.12 Hz, 1H, H-6), 4.08 (ddd, *J* = 6.84 Hz, *J* = 6.12 Hz, *J* = 2.0 Hz, 1H, H-5), 2.46 (t, *J* = 7.74 Hz, 2H, COC*H*_2_CH_2_), 2.30 (t, *J* = 7.56 Hz, 2H, COCH_2_C*H*_2_), 2.12–1.92 (m, 16H), 1.63 (d, *J* = 1.08 Hz, 3H, HC = C(C*H*_3_)_2_), 1.62 (s, 3H), 1.60 (s, 12H) ppm; NMR ^13^C (90.5 MHz, CDCl_3_) *δ*: 174.6 (C, CO), 173.1 (C, C1), 154.0 (C, C3), 136.0 (C, *C*=CH), 135.9 (C, *C*=CH), 135.8 (C, *C*=CH), 134.4 (C, *C*=CH), 132.0 (C, *C*=CH), 126.3 (CH, C=*C*H), 125.6 (CH, C=*C*H), 125.5 (CH, C=*C*H), 125.4 (2CH, C=*C*H), 120.1 (C, C2), 77.1 (CH, C4), 68.0 (CH, C5), 65.7 (CH_2_, C6), 40.9 (CH_2_), 40.8 (CH_2_), 40.7 (CH_2_), 35.7 (CH_2_), 33.9 (CH_2_), 29.2 (2CH_2_), 27.8 (CH_2_), 27.7 (CH_2_), 27.6 (CH_2_), 25.9 (CH_3_, C=C(*C*H_3_)_2_), 17.8 (CH_3_), 16.2 (2CH_3_), 16.1 (CH_3_) , 16.0 (CH_3_) ppm; SM (+APCI) *m/z* (%): 559.6 (100) [M + H]. The IR, ^1^H and ^13^C NMR spectra are presented in Supplementary Figs. S5, S6 and S7, respectively.

#### Preparation of the Vit C–SQ cream

Vit C–SQ (30.1 mg, 0.054 mmol) was weighted in a 5 mL vial. SQ (222 mg, 250 mL, 0.54 mmol) was added via syringe and the mixture was vortexed for 2 min. The obtained mixture was then sonicated using an ultrasonic cleaning bath to give a homogenous cream containing 3 mmol% of Vit C. A similar procedure was followed to obtain the Vit C–SQ cream containing 5 mmol% of Vit C.

#### Formulations for ex vivo skin testing

Explants from two donors aged 30 years were treated with the following formulations: (1) Vit C at 1 or 3 wt% in an aqueous cream of carboxymethyl cellulose (Sigma Aldrich) (2) Vit C–SQ conjugate dispersed in SQ; (3) Vit C–Palmitate complex; (4) squalenic acid in SQ (70:30, w:w) and (5) pure SQ. Final concentrations of Vit C in the various formulations were 1, 3 or 5 wt%.

#### Ex vivo activity testing on human skin

BIO-EC Laboratory possesses an authorization from the Bioethics group of the General Director Services of the French Research and Innovation Ministry (registered under n°DC-2008-542) to use human skin from surgical waste since 5th May 2010. The study was performed in accordance with the Declaration of Helsinki after the patients had given informed consent to use their skin samples by BIO-EC Laboratory.

Skin explants (1-cm diameter) were recovered from an esthetic abdominal surgery in two 30-years old women and maintained in vitro for 9- or 10-days survival at 37 °C, in wet atmosphere enriched with 5% CO_2_, in BIO-EC’s Explants Medium (BEM, BIO-EC Laboratory). At days 0, 1, 3, 6 and 8, explants received topical treatments with each cosmetic formulation (2 mg per explant) spread with a spatula, i.e. free Vit C, Vit C–SQ or Vit C–Palmitate at 1%, 3% or 5% (w:w). As a reference, pure SQ was also tested in one donor (donor 2). Untreated explants sampled at day 10 for donor 1 or 2 showed identical morphology as day-0 explants and were used as controls. Three explant replicates were prepared for each biological condition. Each skin explant was divided in two parts, one was fixed with ordinary Bouin and paraffin-embedded, the other one was frozen at − 80 °C until microdissection and genomic studies were performed.

#### Histological and immunohistochemistry studies

Five-5 µm tissue sections were prepared using a Minot RM 2125 microtome (Leica) and sticked on superfrost silanized glass slides. General tissue morphology was observed after Goldner-modified Masson trichrome staining. Glycosaminoglycans (GAGs) were examined on paraffin-embedded skin sections after staining with Schiff reagent. Neutral GAGs which link growth factors appear as purple pink near the DEJ. Optical microscopy observations were done with an Orthoplan microscope (Leica) at X40 magnification. Pictures were obtained with a tri CCD DXC 390P camera (Sony) and stored with Leica IM1000 software, version 1.10 (Leica). Collagen I immunostaining was done on frozen sections with 800-fold diluted polyclonal rabbit anti-human collagen I antibody (Monosan ref PS047) for one hour at room temperature, with a biotin/streptavidin amplification system, fluorescence revelation (FITC Caltag SA 1001) and nuclei staining with propidium iodide. Collagen III immunostaining was done on formol-fixed and paraffin-embedded slices with 50-fold diluted polyclonal goat anti-human collagen III antibody (SBA ref 1330-01), overnight at room temperature, with a Vectastain RTU Universal VECTOR avidin/biotin amplification system, DAB revelation.

#### Laser capture microdissection

Some skin explants were microdissected in order to separate dermis from epidermis. Serial sections (12-µm-thick human skin frozen tissue specimens) were placed on membrane slides (Carl Zeiss MicroImaging). Cresyl violet staining was performed by using a protocol from Zeiss Labs. Epidermal and dermal tissues were selectively dissected using the PALM MicroBeam system (Carl Zeiss). The dissection procedure was validated by measuring the differential expression of transcripts from genes specifically expressed in each tissue.

#### RNA extraction

Whole skin samples of 40–100 mg weight were homogenized in 1 mL TRI-Reagent (Euromedex), by agitation in a Precellys grinder with MK28-R metallic beads (Bertin) for two cycles of 3 × 20 s at 6500 rpm. After precipitation in 500 µL of isopropanol, RNA was high-speed centrifugated and the RNA pellet was washed twice with 75% ethanol, then solubilized in 30 µL of RNase-free water and stored at − 80 °C until molecular analyses. RNA purity and quantity were assessed by UV absorbance readings and RNA integrity was evaluated by capillary electrophoresis using RNA 6000 Nano chips and the 2100 Bioanalyzer (Agilent Technologies). RNA from microdissection samples were extracted in 300 µL TRI-Reagent, precipitated in 500 µL isopropanol overnight at − 20 °C, solubilized in 20 µL of RNase-free water and quality checked using Total RNA 6000 Pico chips and the 2100 Bioanalyzer. All RNA extracts showed homogeneous quality levels.

#### Amplification and reverse transcription of RNA

Prior to RT-qPCR study, 1 µg of total RNA from whole skin was amplified with the Quick Amp Labeling Kit (Agilent Technologies, ref 5190-0444), consisting of a double strand cDNA synthesis with MMLV-RT and oligo-dT-T7 promoter priming, followed by in vitro transcription with T7 RNA polymerase. Ten microliters of each amplified cRNA was directly reverse transcribed in 20-µL reactions, using Transcriptor First Strand cDNA Synthesis Kit (Roche Diagnostics) and gene-specific priming with a 3-µL mix of qPCR primers designed for all genes studied (0.5 µM of each primer as final concentration in RT). The RNA-primers mix was incubated for 5 min at 65 °C, cooled on ice, completed to 20 µL with RT reagents and incubated for 30 min at 55 °C before enzyme inactivation for 5 min à 85 °C. Similarly, amplification of RNA extracted from each microdissected skin layer was performed from 10 µL of purified RNA solution and then RT was done with a priming mix at 0.07 µM final concentration in RT.

#### Real-time quantitative PCR

PCR primers obtained from the qPrimerDepot database^55^ or designed with Primer Designing Tool (https://www.ncbi.nlm.nih.gov/tools/primer-blast/) were chosen overlapping an intron or hybridizing an exon junction when possible and checked for in silico specificity before synthesis by Eurofins Operon (Supplementary Table S1). qPCR analysis was done in triplicate 15-µL reactions with SYBR Premix Ex Taq Tli RNase H Plus mix (Takara) including 5 µL of 30-fold diluted cDNA and 0.5 µM of each primer. A 2-steps PCR program with hybridization at 61 °C followed by specificity check on fusion curves, was performed on CFX384 thermal cycler (Bio-Rad Laboratories). Quantitative values were normalized using the NORMA-Gene method^56^ for whole-skin data and with *GAPDH* and *ACTB* housekeeping genes for microdissection data^57^. Relative expression ratios were calculated by the delta-delta Cq method^58^, based on a theoretical efficiency considered at 100%, using as calibrator the untreated condition of similar duration as treated samples (day 10).

### Statistical analysis

Data analysis was performed with GraphPad Prism version 5.0.0 for Windows (GraphPad Software, San Diego, California USA, www.graphpad.com). For epidermis and dermis thickness, collagen type I labelling and GAGs staining at DEJ, differences between treatments were tested using one-way ANOVA with Tukey’s multiple comparison post-test, with variances homogeneity checking by Bartlett’s test. For collagen type III labelling and gene expression fold-changes in whole skin explants, differences between treatments were tested using Kruskal–Wallis and post hoc Dunn’s test. For expression data from microdissected skin, differences were tested by two-way ANOVA followed by Bonferroni’s multiple comparison test. Differences between conditions were considered significant when *p* values < 0.05 (*p < 0.05; **p < 0.01; ***p < 0.001). Quantitative data were graphically presented as mean ± standard deviation.

## Supplementary information

Supplementary Information.

Supplementary Dataset.

## Data Availability

The datasets generated and/or analyzed during the current study are provided as Supplementary Data.
