# Supplementary material for: Vitamin C–squalene bioconjugate promotes epidermal thickening and collagen production in human skin
Source: Sci Rep. 2020 Oct 9;10:16883. doi: 10.1038/s41598-020-72704-1 (PMC7547010; doi:10.1038/s41598-020-72704-1)
Supplement: Supplementary file 1 — Supplementary Information. [file 41598_2020_72704_MOESM1_ESM.pdf]

Supplementary Information

Vitamin C–squalene bioconjugate promotes epidermal thickening and collagen production in human skin

R. Gref[1*], C. Deloménie[2], A. Maksimenko[3], E. Gouadon[4], G. Percoco[5], E. Lati[5,6], D. Desmaële[3], F. Zouhiri[3], P. Couvreur[3*]

[1] *Institut des Sciences Moléculaires d'Orsay, UMR 8214 CNRS, Université Paris-Saclay, 91405 Orsay, France.*

[2] *UMS-IPSIT – Université Paris-Saclay, 5 rue Jean-Baptiste Clément 92296 Châtenay-Malabry, France.*

[3] *UMR/CNRS 8612 Institut Galien Paris-Saclay, Faculté de Pharmacie, Université Paris-Saclay, 5 rue Jean-Baptiste Clément, 92296 Châtenay-Malabry, France.*

[4] *UMR-S 999, LabEx LERMIT, Centre Chirurgical Marie Lannelongue, 133 avenue de la Résistance, 92350 Le Plessis-Robinson, France.*

[5] *Laboratoire BIO-EC, 91160, Longjumeau, France.*

[6] *GENEX, 91160, Longjumeau, France.*

Supplementary Methods

Supplementary Table S1. Primers used for RT-qPCR.

Gene symbol	Forward primer	Reverse primer	Product length (bp)
ACTB	AATGATGAGCCTTCGTG	AACTGGTCTCAAGTCAGTGT	130
ADIPOQ	CCATCTCCTCCTCACTTCCA	TAGAACAGCTCCCAGCAACA	91
B3GALT6	GCAGTGGTCTCAGGAGGAAG	TTTGGAGGTGGGAGAATCAC	95
COL1A1	TGTTCAGCTTTGTGGACCTC	CTGTACGCAGGTGATTGGTG	125
COL1A2	GAAAAGGAGTTGGACTTGGC	AGCAGGTCCTTGGAAACCTT	104
COL3A1	GGATTCTCCTCCTTCATCCTGTAA	TTCAACACATTCTCTATGCTAGTGT	89
DCN	TCAGCTATTTCTTCTACCTCTCCCT	AGACCAGTCAGGAAATGTATGCT	110
DSE	ATGGCAGCGCAGCCTAGTT	GGCAAAACCAACCAGGGAGT	81
FBN1	GTGAATGTACAAACACAGTCAGCA	AACAGTATCCTGGGCGAACA	105
GAPDH	CAGCCTCAAGATCATCAGCA	TGTGGTCATGAGTCCTTCCA	106
HAS2	GACAGGCTGAGGACGACTTT	TCCAAAGAGTGTGGTTCCAA	97
HMMR	TGCAGCTCAGGAACAGCTAA	GCTGACAGCGGAGTTTTGAT	149
LAMA5	AAGGTGCACTGGGTGTATCA	TCTGTCTCTCCACTGTGACG	142
MMP1	TTCGGGGAGAAGTGATGTTC	TTGTGGCCAGAAAACAGAAA	101
PPIB	GTTTGAAGTTCTCATCGGGG	AAAACAGCAAATTCCATCGTG	123
TIMP1	CTGTTGTTGCTGTGGCTGAT	TCTGGTTGACTTCTGGTGTCC	130
TNXB	GGACAGAGCAGGTGCAGAG	GAACCAGGCTGGAGGTTAGA	92

Supplementary Results

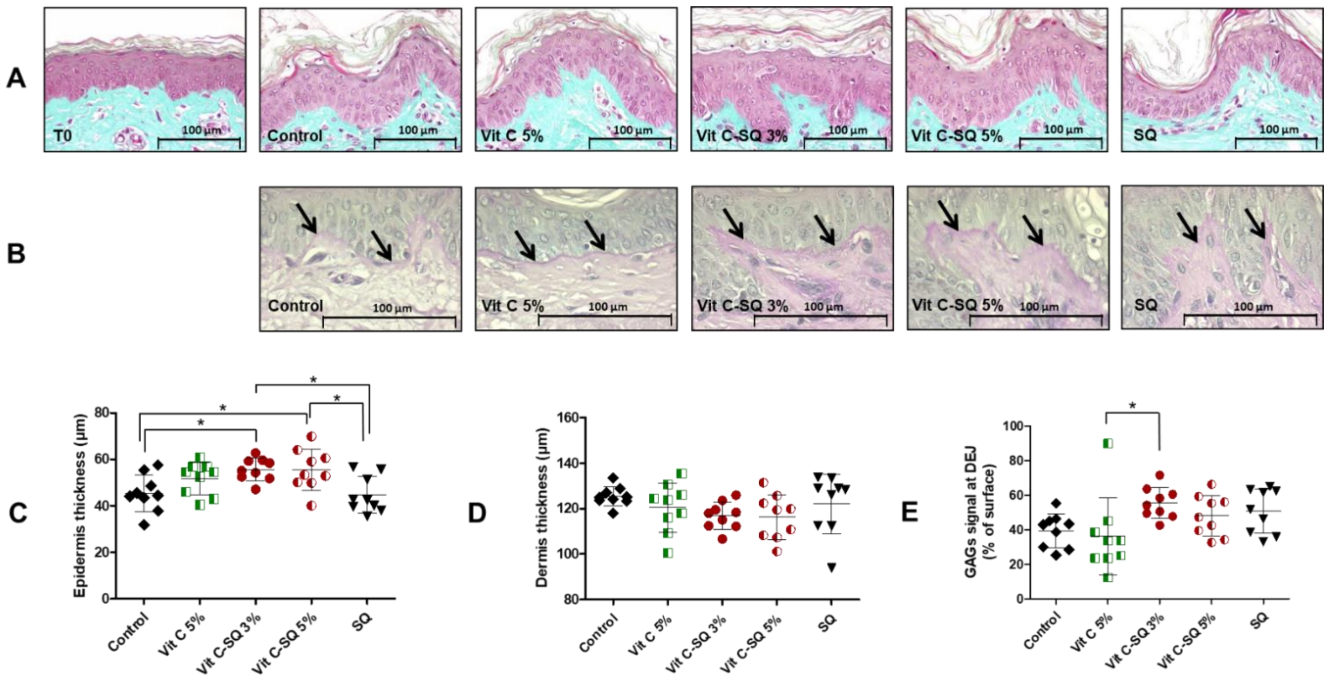

Supplementary Figure S1: Topical treatment of human skin explants from donor 2 for 10 days with 4 formulations containing either free Vit C at 5 wt% or Vit C-SQ at or 3 or 5 wt%, or SQ alone, modified epidermis thickness and GAGs content at dermal-epidermal junction (DEJ). (**A**) In whole skin sections fixed and stained by Masson trichrome, epidermis (pink) and dermis (blue) layers were observed before (T0) and after the treatment period. The control explant was kept untreated for 10 days. (**B**) Skin sections stained with Schiff reagent revealed the GAGs as purple pink near DEJ (arrows). Pictures were realized with a tri CCD DXC 390P camera (Sony) and stored with Leica IM1000 software, version 1.10 (www.leica-microsystems.com). (**C**) The epidermis thickness and (**D**) dermis thickness were measured in treated and control explants (n=9 images; mean of 3 measures per image). (**E**) The intensity GAGs staining at DEJ was measured as the percentage of surface (n=3 images; mean of 3 measures per image). One-way ANOVA with Tukey's post-test was used to compare treatments (*$p < 0.05$). Graphs were done with GraphPad Prism version 5.0.0 for Windows (GraphPad Software, San Diego, California USA, www.graphpad.com). Scale bar = 100 µm.

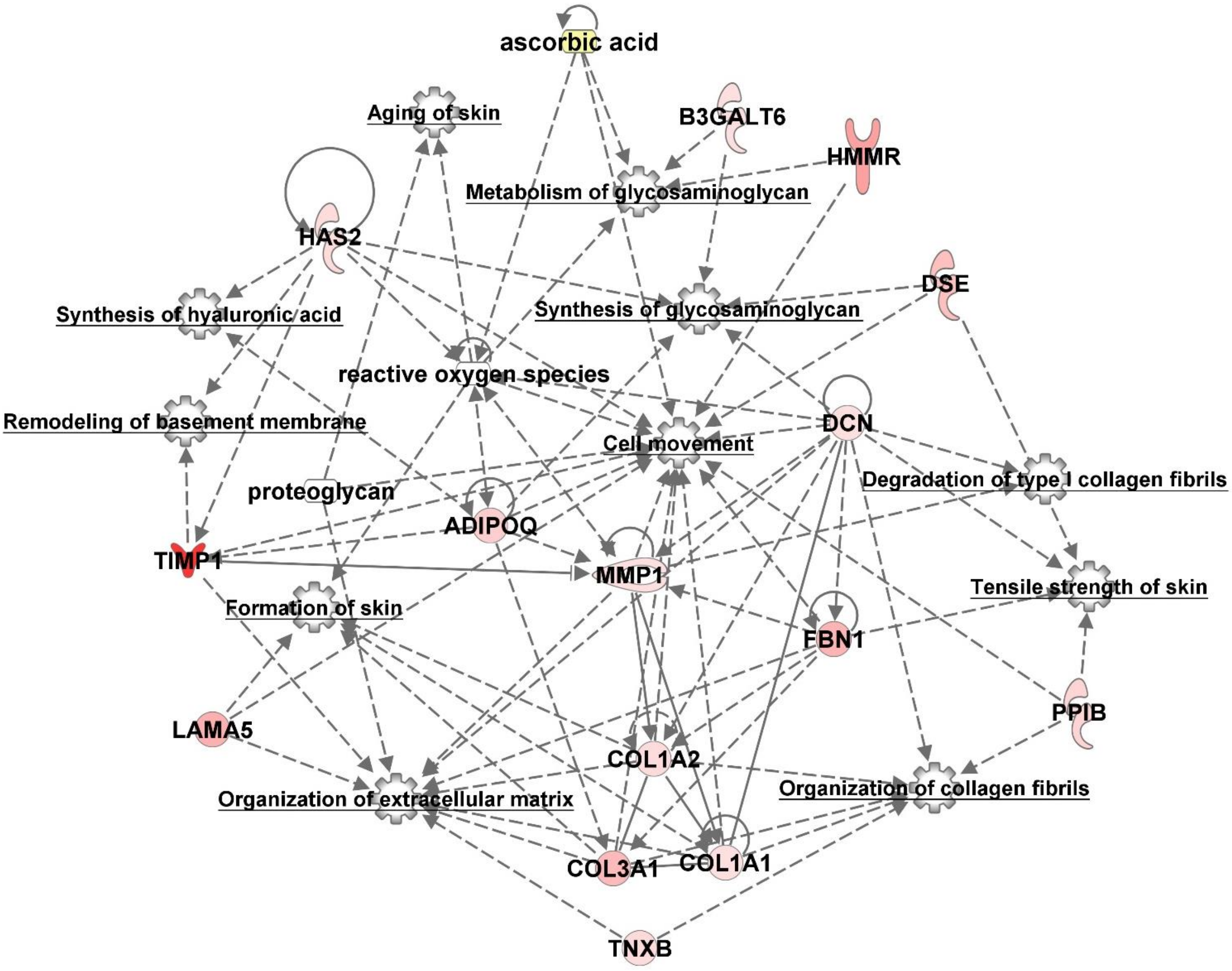

Supplementary Figure S2: The relationships between genes studied, molecules including Vitamin C and known biological pathways relevant in skin physiopathology (underlined) were documented and the network was generated through the use of IPA (QIAGEN Inc., https://www.qiagenbio-informatics.com/products/ingenuity-pathway-analysis). For genes, the color code represents the mean fold-change observed in whole skin from donor 1 after treatment with Vit C-SQ 3 wt% compared to untreated control (red intensity reflects the strength of observed up-regulation).

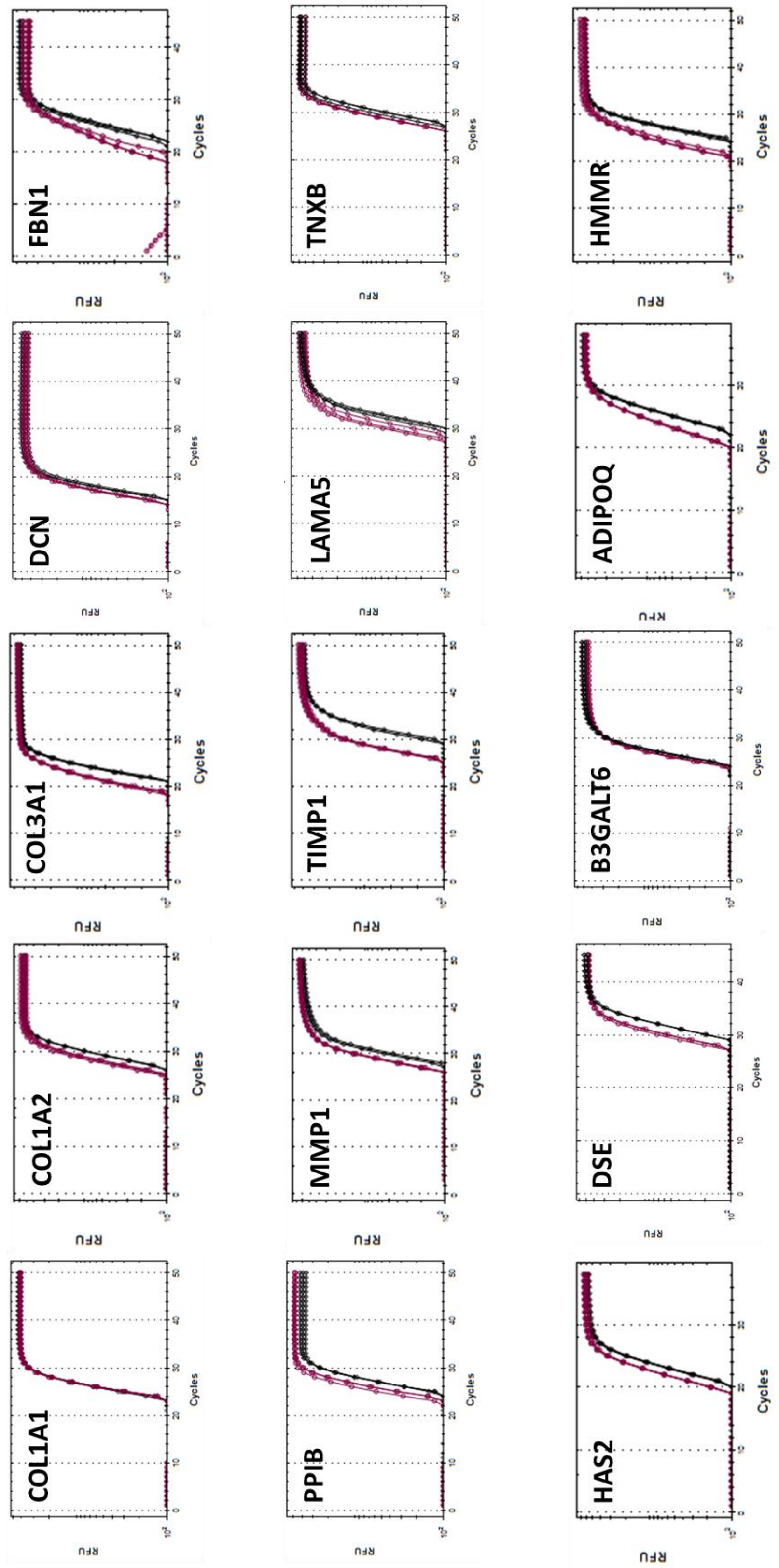

Supplementary Figure S3: Raw qPCR data showing the relative expression of the 15 studied genes in whole skin from donor 1 after treatment with Vit C-SQ 3 wt% (red ●) compared to untreated control (black ◆). The curves were provided by the CFX Manager software version 3.1 (Bio-Rad Laboratories, USA, https://www.bio-rad.com/).

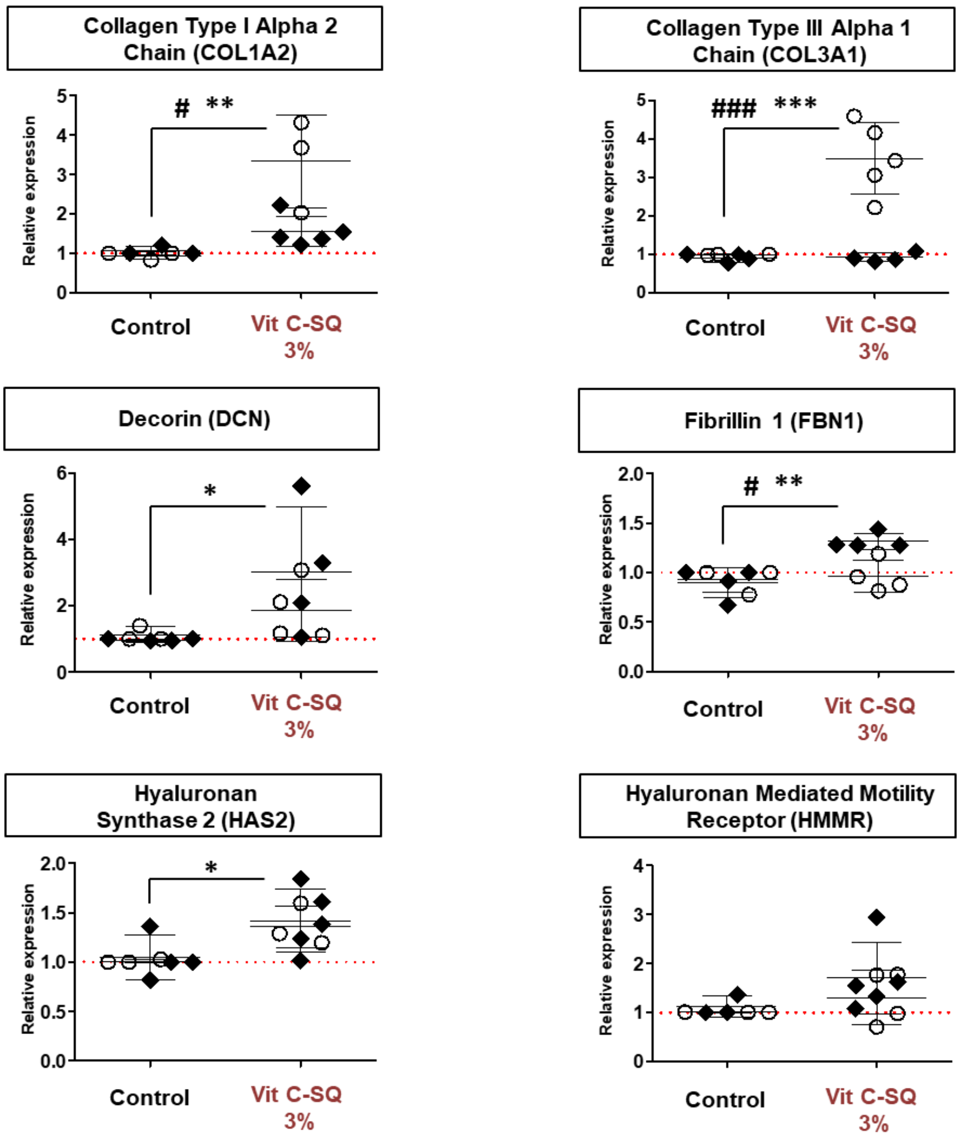

Supplementary Figure S4: Vitamin C-Squalene could differentially influence in dermis and epidermis the expression of genes either encoding collagens (*COL1A2*, *COL3A1*), or controlling skin fibrils assembly (*DCN*, *FBN1*) or GAGs biosynthesis (*HAS2*) or cell growth and motility (*HMMR*). Measures were performed from 3 to 5 dermis (○) or epidermis (◆) samples microdissected in tissue sections prepared from human skin explants, each sample totalizing a surface of 1.4 to 5.3 μm^2 of dissected material. Fold-changes of each mRNA transcript were calculated as $2^{-\Delta\Delta Cq}$ with normalization upon *ACTB* and *GAPDH* transcripts, relative to untreated control (set to 1, red dotted line). Data were pooled from donor 1 and 2 skin explants which both received the treatment by Vit C-SQ 3 wt% formulation. Two-way ANOVA followed by Bonferroni's post-test was used to determine significance between the groups regarding the effect of tissue (### $p < 0.001$, # $p < 0.05$) or the effect of treatment (***$p < 0.001$, **$p < 0.01$, *$p < 0.05$). Graphs were done with GraphPad Prism version 5.0.0 for Windows (GraphPad Software, San Diego, California USA, www.graphpad.com).

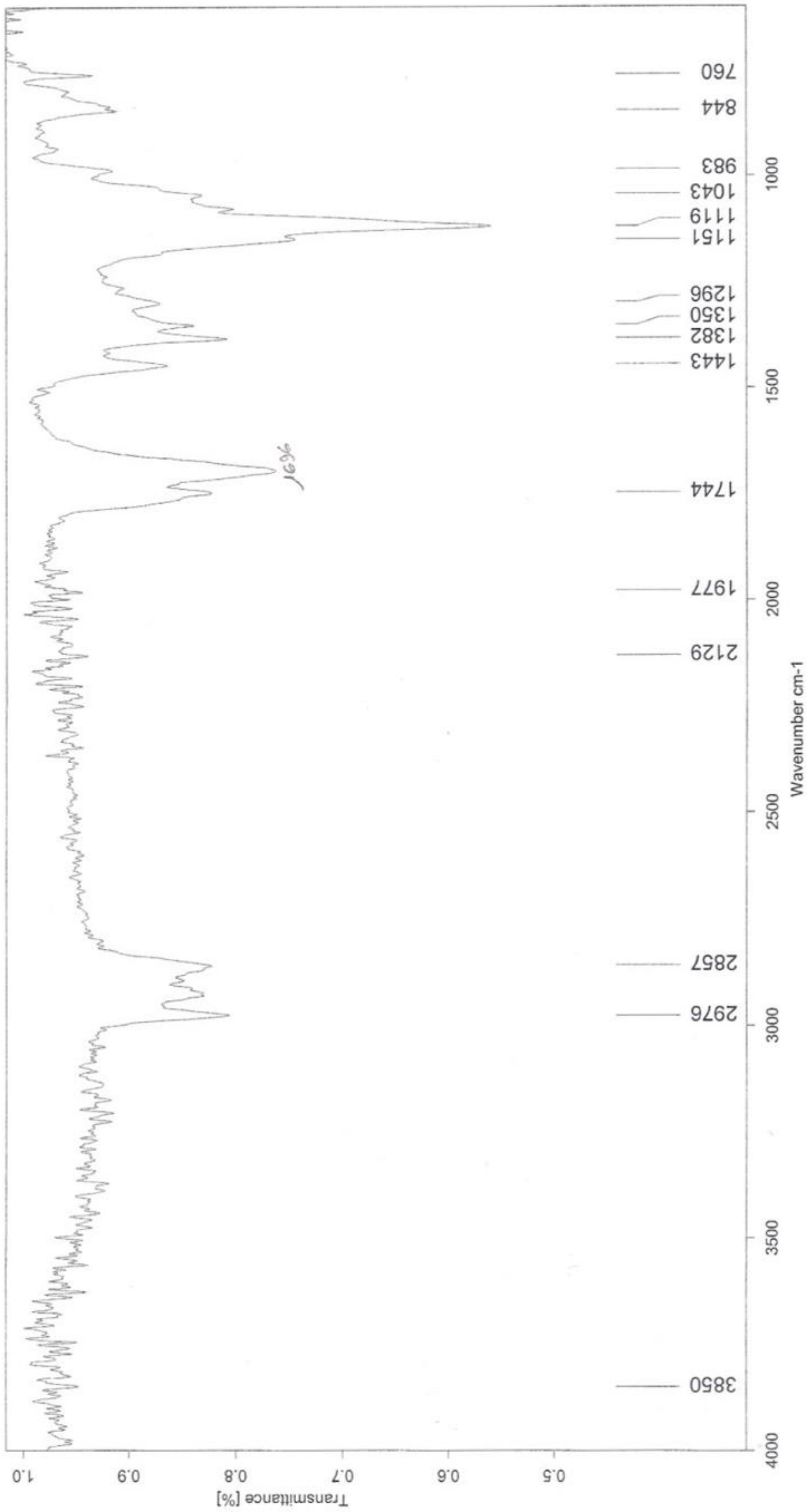

Supplementary Figure S5: IR spectrum of Vit C-SQ.

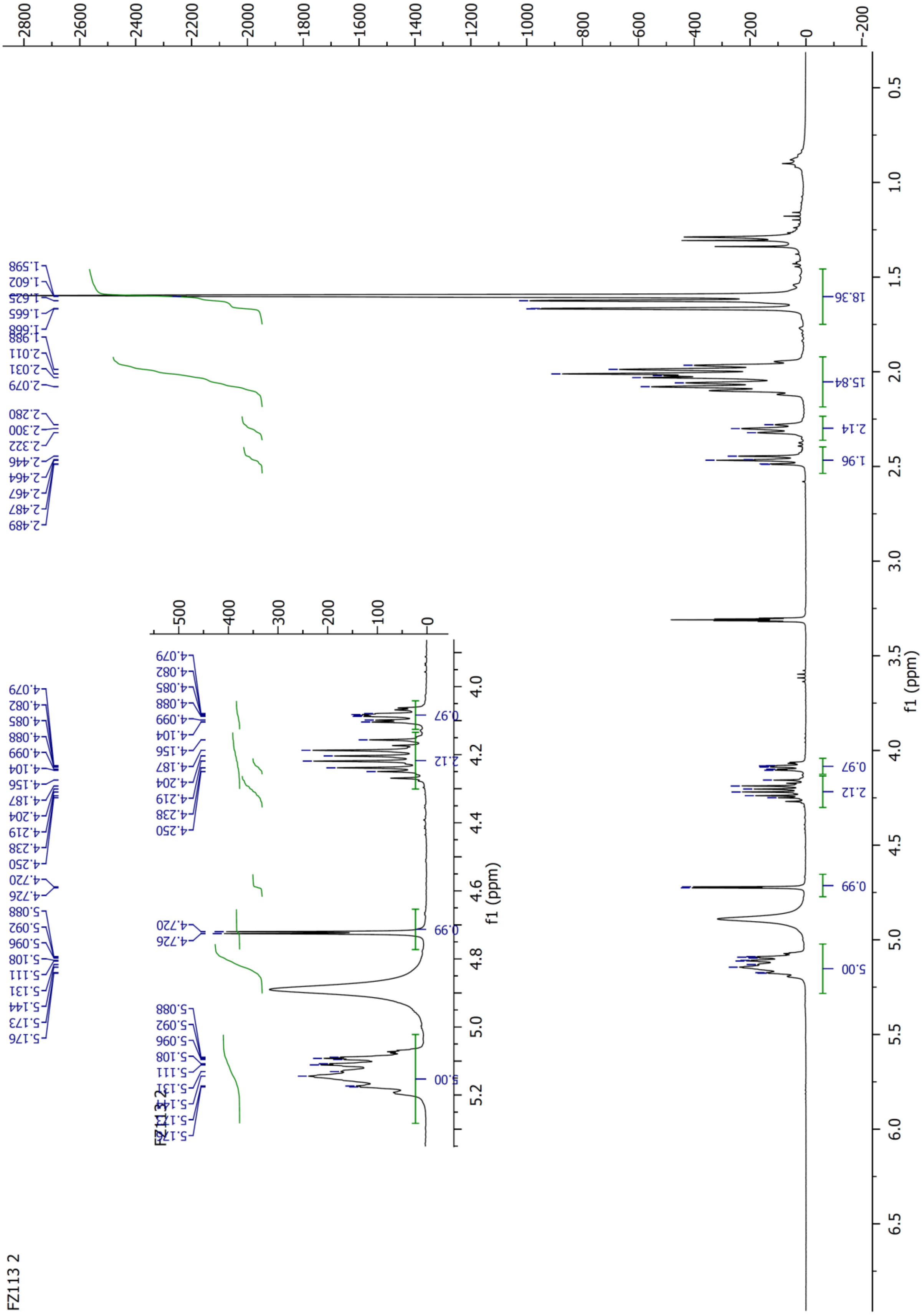

Supplementary Figure S6: ^{1}H NMR of Vit C SQ.

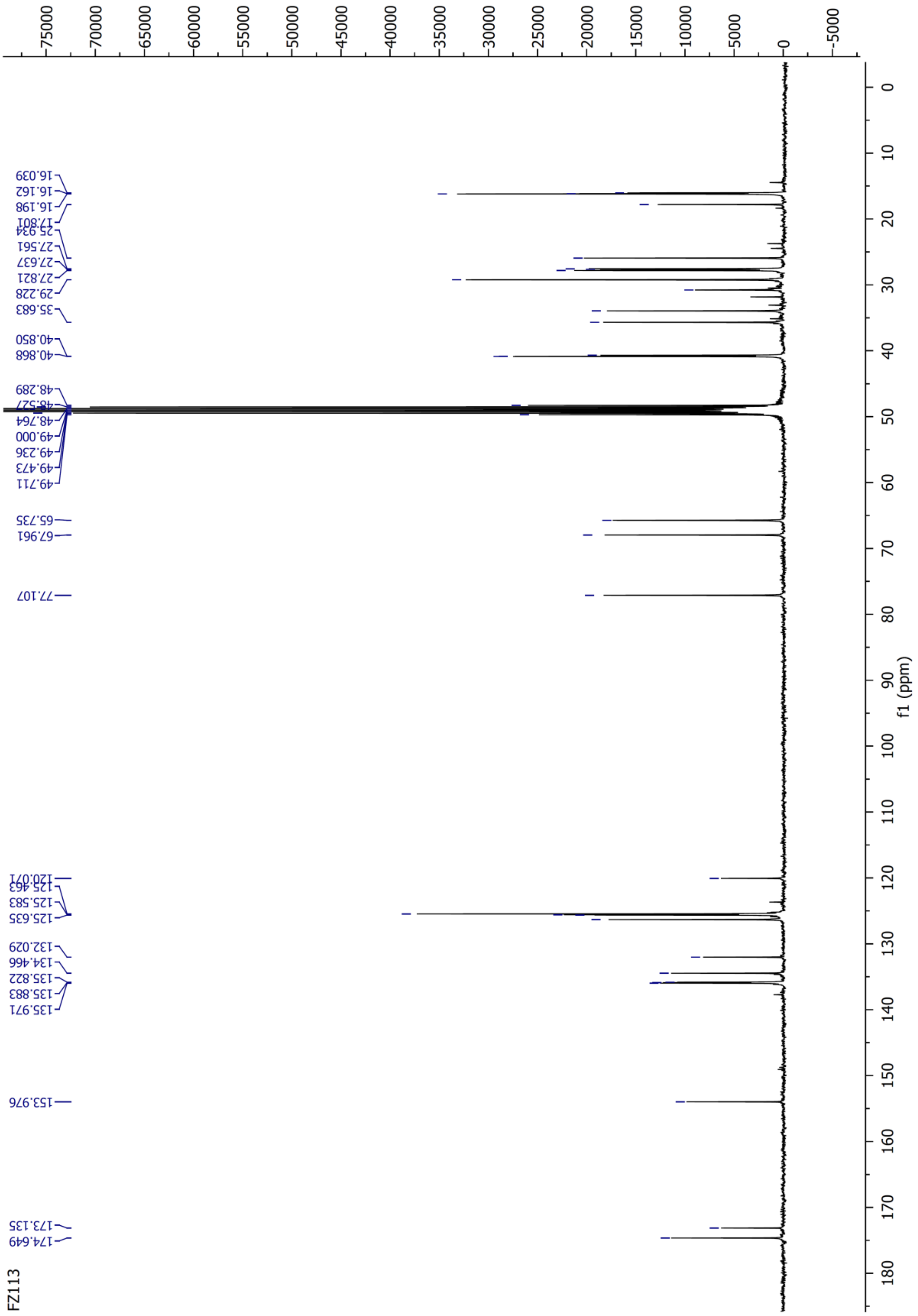

Supplementary Figure S7: ^{13}C NMR of Vit C SQ.
